# Exogenous Gibberellin Treatment Enhances Melatonin Synthesis for Melatonin-Enriched Rice Production

**DOI:** 10.3390/biom12020198

**Published:** 2022-01-24

**Authors:** Ok Jin Hwang, Kyoungwhan Back

**Affiliations:** Department of Biotechnology, College of Agriculture and Life Sciences, Chonnam National University, Gwangju 61186, Korea; smilax@chonnam.ac.kr

**Keywords:** gibberellins, *GA3ox2*, G protein alpha, melatonin, RNAi transgenic rice

## Abstract

Melatonin production is induced by many abiotic and biotic stressors; it modulates the levels of many plant hormones and their signaling pathways. This study investigated the effects of plant hormones on melatonin synthesis. Melatonin synthesis in rice seedlings was significantly induced upon exogenous gibberellin 3 (GA_3_) treatment, while it was severely decreased by GA synthesis inhibitor paclobutrazol. In contrast, abscisic acid (ABA) strongly inhibited melatonin synthesis, whereas its inhibitor norflurazon (NF) induced melatonin synthesis. The observed GA-mediated increase in melatonin was closely associated with elevated expression levels of melatonin biosynthetic genes such as *TDC3*, *T5H*, and *ASMT1*; it was also associated with reduced expression levels of catabolic genes *ASDAC* and *M2H*. In a paddy field, the treatment of immature rice seeds with exogenous GA led to enhanced melatonin production in rice seeds; various transgenic rice plants downregulating a GA biosynthesis gene (*GA3ox2*) and a signaling gene (*Gα*) showed severely decreased melatonin levels, providing in vivo genetic evidence that GA has a positive effect on melatonin synthesis. This is the first study to report that GA is positively involved in melatonin synthesis in plants; GA treatment can be used to produce melatonin-rich seeds, vegetables, and fruits, which are beneficial for human health.

## 1. Introduction

Melatonin is an indole alkaloid commonly found in plants and animals. In plants, melatonin is involved in normal growth and development, and defense responses against many biotic and abiotic stressors [1,2]. The mode of action of melatonin in plant growth and development is presumably associated with the interactions of various plant hormones, including gibberellins (GAs), ethylene, and brassinosteroids (BRs) [1]. The protective effects of melatonin against multiple adverse environmental (e.g., biotic and abiotic) stresses are attributable to its potent antioxidant and regulatory activities, which induce a vast array of relevant genes [2,3]. A recent study showed that melatonin acts as a signaling molecule in the regulation of starch synthesis during normal growth [4]; it also functions in the regulation of molecular chaperones in response to heat stress [5]. The involvement of melatonin in a diverse array of biological activities strongly suggests that, similar to animals, plants possess melatonin receptors [6]. Although Cand2 was recently proposed as a phytomelatonin receptor [7], its potential phytomelatonin receptor role is controversial [8].

The extremely low levels of melatonin during normal plant growth and development [9] support a role for melatonin as a necessary signaling molecule for phytomelatonin receptor function in plants. For example, rice produces melatonin at a rate of ca. 0.5 ng/g fresh weight (FW) [10], whereas *Arabidopsis* and cassava produce melatonin at rates of 0.05 and 0.006 ng/g FW, respectively [11,12]. The low levels of melatonin in plants are consistent with the low enzymatic activities of key melatonin biosynthetic enzymes such as serotonin *N*-acetyltransferase (SNAT) and *N*-acetylserotonin *O*-methyltransferase (ASMT) [13]. However, melatonin is induced in response to many stress conditions; it helps to protect plants from stress damage [14,15]. For example, melatonin in rice leaves increased from 0.5 to 225 ng/g FW upon cadmium challenge [16]. Melatonin affects many plant hormones, including auxin, GA, cytokinins, abscisic acid (ABA), ethylene, salicylic acid, jasmonates, and BR under normal conditions and under various stresses [1]; despite investigations thus far, the role of plant hormones in the regulation of melatonin synthesis remains poorly understood. The effects of melatonin on plant hormones vary among plant species, which suggests complex regulatory networks linking melatonin and plant hormones. As our initial study of the roles of plant hormones in melatonin synthesis, we focused on GA because *Arabidopsis thaliana SNAT1* and *SNAT2* knockout mutants (*snat1* and *snat2*) showed delayed flowering [4,17] in response to a significant decrease in *ent-kaurene synthase* (*KS*), the key gene for GA synthesis [17]. Melatonin treatment increases GA content in some plant species, including cucumber, cotton, rapeseed, apple, and pear [1]. Rice has long been used as a model plant to study melatonin because rice synthesizes melatonin at a rate of up to 0.5 ng/g FW, which can be easily measured using high-performance liquid chromatography (HPLC), and because melatonin production increases dramatically in response to cadmium treatment. These factors facilitate the investigation of melatonin synthesis in rice plants. The goal of this study was to identify plant hormones that can modulate melatonin synthesis, especially for hormones for inducing melatonin synthesis to produce melatonin-enriched plants or fruits, which are beneficial to human health.

## 2. Materials and Methods

### 2.1. Plant Growth Conditions

Rice (*Oryza sativa* cv. Dongjin) seeds were sterilized and grown on half-strength Murashige and Skoog medium under cool daylight fluorescent lamps (60 μmol m^−2^ s^−1^; Philips, Amsterdam, Netherlands) with a 14 h light/10 h dark photoperiod (28/24 °C). Germinated seeds were transplanted into soil as a field test. The plants were grown in a paddy field at Chonnam National University (35°09′ N, 126°54′ W; 53 m a.s.l.), Gwangju, Korea. To investigate the effects of GA on melatonin accumulation in rice seeds, commercially available GA_4+7_ solution (100 μM; Daeyu Co., Ltd., Seoul, Korea) was sprayed with handheld garden sprayer (Kyeyang Co., Cheongju city, Korea) onto immature rice seeds at various time intervals (three applications, once every other day) beginning 1 week after flowering. At 60 days after flowering (DAF) which was about 180 days after sowing, rice seeds were harvested for melatonin quantification. Each treatment was replicated three times.

### 2.2. Chemical Treatment

Seven-day-old rice seedlings in 50 mL polypropylene conical tubes containing 30 mL water were rhizospherically pretreated with ABA (1 μM; Sigma-Aldrich, St. Louis, MO, USA), norflurazon (10 μM; Sigma-Aldrich), GA_3_ (10 μM; Duchefa Biochemie, Haarlem, The Netherlands), paclobutrazol (10 μM; Sigma-Aldrich), or 1% ethanol (mock control). Pretreatments were applied for 24 h; seedlings were then transferred into new conical tubes containing 0.5 mM CdCl_2_ for 3 days under continuous light (60 μmol photons m^−2^ s^−1^). Leaves and stems were harvested for melatonin quantification.

### 2.3. Quantitative Real-Time Polymerase Chain Reaction (qPCR) Analysis

Total RNA of the rice plants was isolated using a NucleoSpin RNA Plant Kit (Macherey-Nagel, Düren, Germany). First-strand cDNA was synthesized from 2 μg of total RNA using MG MMLV Reverse Transcriptase (MGmed, Inc., Seoul, Korea) and an oligo dT_18_ primer at 42 °C for 1 h. qPCR was performed in a Mic qPCR Cycler system (Biomolecular Systems, Upper Coomera, QLD, Australia) with specific primers and the Luna Universal qPCR Master Mix (New England Biolabs, Ipswich, MA, USA), as described previously. Gene expression was analyzed using Mic RQ software v2.2 (Biomolecular Systems) and normalized to *ACT1*. Reverse transcription (RT)-PCR and qPCR were performed using the following primer set: *GA3ox2* forward 5′-CTT GAA GAA CCC GCT CTG-3′, *GA3ox2* reverse 5′-GAA ACT CCT CCA CAC GTC ACA-3′; *G-alpha* forward 5′-GAA ACT CCT CCA TCA CGT CAC A-3′, *G-alpha* reverse 5′-CAT CGT CAC GCA TCT CAG-3′; *UBQ5* forward 5′-CCG ACT ACA ACA TCC AGA AGG AG-3′, *UBQ5* reverse 5′-AAC AGG AGC CTA CGC CTA AGC-3′; *TDC1* forward 5′-GGC TCA AGC TCT GGA TGG TCA TG-3′, *TDC1* reverse 5′-GCG AAG TTC CTC GGC ACG AC-3′; *TDC2* forward 5′-ATG CCC AGA GTA CCG ACA CC-3′, *TDC2* reverse 5′-CCT TAA CCC ATA GCA AGG AAC AA-3′; *TDC3* forward 5′-GTG GCT AAA ACA TCT CGG TAG G-3′, *TDC3* reverse 5′-GCA GGA TTA TTT TGC CGT GTC-3′; *T5H* forward 5′-CCT CGT CCT GGA CAT GTT CGT C-3′, *T5H* reverse 5′-ATG GCG AAC GTG TTG ATG AAC AC-3′; *SNAT1* forward 5′-CAG TAG AGC CAC CAT CAG CA-3′, *SNAT1* reverse 5′-ATC CCA CCT TGT CGC ATA AA-3′; *SNAT2* forward 5′-GTC TGG GAC GTG GTC GTG-3′, *SNAT2* reverse 5′-GTT GCC TTG AGC GGT AGA AG-3′; *COMT* forward 5′-CCT GCT CGC CTC CTA CAA-3′, *COMT* reverse 5′-ATG CCC TCG TTG AAG ACG-3′; *ASMT1* forward 5′-GCC AAG GCT CCC AGT AAC AA-3′, *ASMT1* reverse 5′-CCT TTC CTC CAG CAT CCC TC-3′; *ASDAC* forward 5′-ATG GAA CAG CTG TGG G-3′, *ASDAC* reverse 5′-ACC ACG ATG CTT CGA AGT-3′; *M2H* forward 5′-ACT AGT ATG CCC GCC GTG GCC-3′, *M2H* reverse 5′-GAG CTC GTG TCG TAC CTG-3′; *M3H* forward 5′-ACT AGT ATG GCG GGA GCA AGA-3′, *M3H* reverse 5′-GAG CTC GCT TTT AGT CTC TGA-3′; and *ACT1* forward 5′-TGC TAT GTA CGT CGC CAT CCAG-3′, *ACT1* reverse 5′-AAT GAG TAA CCA CGC TCC GTCAA-3′.

### 2.4. Quantification of Serotonin, N-Acetylserotonin, and Melatonin 

Frozen samples (0.1 g) were pulverized into a powder in liquid nitrogen using the TissueLyser II (Qiagen, Tokyo, Japan) and then extracted with 1 mL methanol for serotonin and *N*-acetylserotonin quantification, and with 1 mL chloroform for melatonin quantification. Prior to serotonin and *N*-acetylserotonin measurements, methanol extracts were centrifuged for 10 min at 12,000× *g*; supernatants (10 µL) were subjected to HPLC using a fluorescence detector system (Waters, Milford, MA, USA). Prior to melatonin measurement, chloroform extracts were centrifuged for 10 min at 12,000× *g*, and resulting supernatants (200 µL) were completely evaporated and dissolved in 0.1 mL of 40% methanol; 10 µL aliquots were subjected to HPLC using a fluorescence detector system (Waters), as described previously [16]. All measurements were performed in triplicate.

### 2.5. Generation of GA3ox2- and Gα-Suppression Transgenic Rice Plants via RNA Interference (RNAi)

The pTCK303 binary vector was used to suppress either the *GA3ox2* (GenBank accession number Os01g0177400) or *Gα* rice gene (GenBank accession no. D38232), as previously described [18]. Briefly, an *N*-terminal 130 bp *GA3ox2* cDNA fragment was amplified by RT-PCR using the following primer set: *GA3ox2-F* 5′-ACT AGT TCC TCC TTC TTC TCC AAG-3′ (*Spe*I site underlined) and *GA3ox2-R* 5′-GAG CTC AAA CTC CTC CAT CAC GTC ACA-3′ (*Sac*I site underlined) with the cDNA templates synthesized from total RNA from rice seedlings. For *Gα*, a *C*-terminal 267-bp *Gα* cDNA fragment was amplified using the following primer set: *Gα-F* 5′-ACT AGT AGC GAA TAT GAT CAG ATG CTA-3′ (*Spe*I site underlined) and *Gα-R* 5′-GAG CTC TTC AAA CTT CTT CTT GAC-3′ (*Sac*I site underlined). Both PCR products were subcloned into the T and A cloning vector (T and A:GA3ox2 and T and A:Gα; RBC Bioscience, New Taipei City, Taiwan) prior to additional cloning procedures. From both T and A:GA3ox2 and T and A:Gα plasmids, the antisense *GA3ox2* and *Gα* inserts were prepared by *Sac*I and *Spe*I double digestion; sense *GA3ox2* and *Gα* inserts were prepared by *Kpn*I and *Bam*HI double digestion. The antisense fragments were first ligated into the pTCK303 vector, followed by the sense fragments of both inserts. The resulting pTCK303:GA3ox2 and pTCK303:Gα RNAi binary vectors were independently transformed into *Agrobacterium tumefaciens* LBA4404; *Agrobacterium*-mediated rice transformation was performed using embryogenic calli derived from *O. sativa* cv. Dongjin rice seeds, as previously described [8,19].

### 2.6. Statistical Analyses

All data were analyzed using analysis of variance (ANOVA) with IBM SPSS Statistics 23 software (IBM Corp., Armonk, NY, USA). Means with different letters or asterisks indicate significantly different values evaluated using *p* values < 0.05, according to the least significant difference test or Tukey’s post hoc honest significant difference (HSD) test. All data are presented as means ± standard deviations.

## 3. Results

### 3.1. GA Pretreatment Increases Melatonin Synthesis in Response to Cadmium

**Figure 1:**
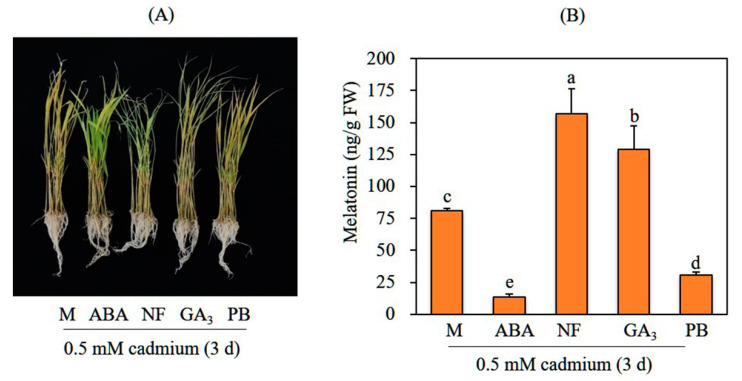
Melatonin content in response to plant hormones. (**A**) Phenotypes of 7-day-old rice seedlings after hormone and cadmium treatments. (**B**) Melatonin content. Seven-day-old rice seedlings rhizospherically treated with various hormones independently for 24 h, then treated with 0.5 mM cadmium for 3 days. Different letters indicate significant differences (*p* < 0.05; analysis of variance (ANOVA), followed by Tukey’s honest significant difference (HSD) post hoc tests). M, water containing 0.1% ethanol; ABA, 1 μM abscisic acid; NF, 10 μM norflurazon; GA_3_, 10 μM gibberellic acid 3; PB, 10 μM paclobutrazol.

To examine the effects of GA on melatonin synthesis, 7-day-old rice seedlings were rhizospherically pretreated GA_3_, followed by cadmium treatment to induce melatonin induction. A GA concentration of 10 μM GA is commonly used in GA assays for germination and third leaf sheath elongation tests [20]. Melatonin was produced at rates of ca. 76 ng/g FW by mock control rice seedlings and 125 ng/g FW by GA_3_-pretreated rice seedlings, representing a 1.8-fold difference (Figure 1). In contrast, pretreatment with 10 μM of paclobutrazol, a GA biosynthesis inhibitor, sharply inhibited cadmium-induced melatonin synthesis, such that rice seedlings produced threefold less melatonin than the mock control did (25 ng/g FW); this indicated the involvement of GA in cadmium-induced melatonin biosynthesis. In sharp contrast, ABA pretreatment (1 μM) resulted in the lowest melatonin production (12 ng/g FW), sixfold less than that of the mock control. To confirm an inhibitory effect of ABA on melatonin synthesis, rice seedlings were pretreated with the ABA biosynthesis inhibitor norflurazon and challenged with cadmium. Rice seedlings pretreated with 10 μM norflurazon exhibited the highest melatonin production (160 ng/g FW), indicating that ABA is a potent melatonin synthesis inhibitor. In summary, GA is a potent melatonin synthesis inducer, whereas ABA is a potent melatonin synthesis inhibitor.

### 3.2. GA Dose-Dependent Melatonin Production

**Figure 2:**
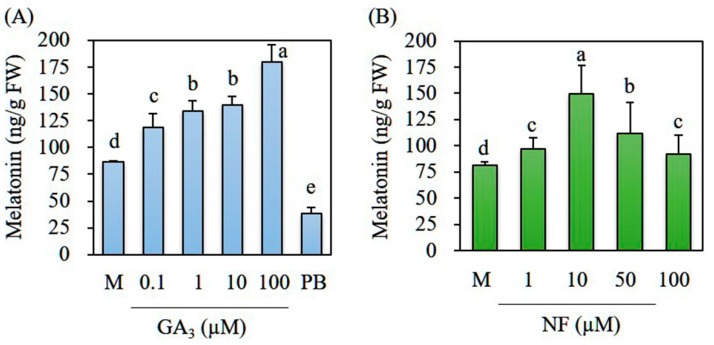
Dose-dependent melatonin levels in response to (**A**) GA_3_ and (**B**) norflurazon. Various concentrations of GA_3_ or NF were used to rhizospherically treat to 7-day-old rice seedlings for 24 h, followed by treatment with 0.5 mM cadmium for 3 days. Melatonin content was quantified using high-performance liquid chromatography (HPLC). Different letters denote significant differences (*p* < 0.05; ANOVA, followed by Tukey’s HSD post hoc tests). M, water containing 0.1% ethanol; GA_3_, gibberellic acid 3; PB, pachlobutrazol; NF, norflurazon.

To determine the optimal GA_3_ concentration for melatonin induction, we pretreated 7-day-old rice seedlings with various concentrations of GA_3_ (0.1–100 μM) for 24 h. The resulting seedlings were challenged with cadmium for 3 days. We observed a GA dose-dependent increase in melatonin production (Figure 2A). Even in the 0.1 μM GA_3_ pretreatment, melatonin synthesis was significantly increased, with a peak of 180 ng/g FW melatonin in the 100 μM GA_3_ treatment. In contrast, paclobutrazol treatment (10 μM) inhibited melatonin induction. Norflurazon treatment showed no dose-dependent increase in melatonin (Figure 2B); melatonin synthesis increased, peaked, and decreased at norflurazon treatment levels of 1, 10, and 50 μM, respectively. At a 100 μM dose of norflurazon, melatonin synthesis was comparable with synthesis in the mock control. These adverse effects of high norflurazon concentration on melatonin synthesis may be ascribed to its inhibition of carotenoid biosynthesis, a key pigment for photosynthesis [21,22]. Because melatonin induction under cadmium treatment requires light and its receptor phytochrome [23], pigment disruption appears to inhibit light absorption that is essential to melatonin induction.

### 3.3. Increased Melatonin Production in Rice Seedlings and Seeds after GA Treatment in the Absence of Cadmium Treatment

**Figure 3:**
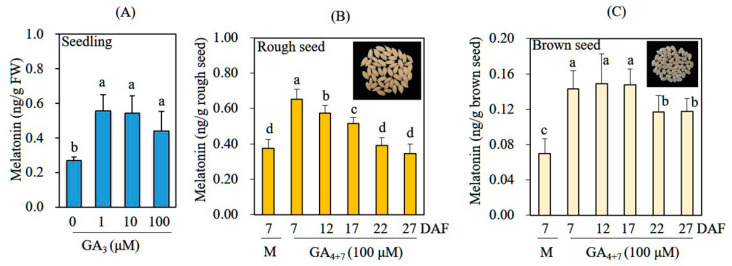
Melatonin levels in (**A**) rice seedlings, (**B**) rough seeds, and (**C**) brown seeds after treatment with GA in the absence of cadmium treatment. Seven-day-old rice seedlings were treated with various concentrations of GA_3_ for 24 h; leaves or seeds were subjected to HPLC quantification of melatonin. In addition, immature rice seeds were treated at various time intervals with 100 µM of commercially available GA_4+7_ (Daeyu Co., Ltd.) three times every other day in the field. After 60 days after flowering (DAF), rice seeds were harvested for melatonin quantification. Different letters denote significant differences (*p* < 0.05; ANOVA, followed by Tukey’s HSD post hoc tests). M, water containing 0.1% ethanol; GA_3_, gibberellic acid 3.

To investigate whether GA treatment enhances melatonin production in rice seedlings and rice seeds in the absence of cadmium treatment, we rhizospherically treated 7-day-old rice seedlings with various concentrations of GA_3_ for 24 h and then performed melatonin quantification. Leaves of the mock control produced 0.25 ng/g FW melatonin, whereas 1 μM GA_3_-treated leaves produced melatonin at a rate of 0.55 ng/g FW (Figure 3A). A dose-dependent increase in melatonin production was not observed under increasing GA_3_ concentrations, in contrast to our results for cadmium-treated leaves (Figure 2A). To determine whether GA treatment could also induce melatonin production in seeds, we sprayed immature rice seeds grown in the paddy field with 100 μM GA_4+7_, beginning at 7 DAF, at various time intervals. At 60 DAF, rice seeds were harvested for melatonin quantification. Untreated control rice seeds contained 0.35 ng melatonin/g rough seed, whereas GA-treated seeds at 7 DAF contained 0.67 ng melatonin/g rough seed; this represented a nearly twofold increase (Figure 3B). Melatonin levels gradually decreased in rice seeds when GA was supplied later than 7 DAF. Among husked (brown) rice seed, control rice seeds contained 0.07 ng melatonin/g brown seed, whereas GA-treated rice seeds produced 0.14 ng melatonin/g brown seed; this represented a twofold increase. Collectively, these data indicate that GA elicits melatonin synthesis in both leaves and seeds of rice plants. 

### 3.4. Characterization of Genes Involved in Melatonin Biosynthesis and Catabolism in Response to GA Treatment

**Figure 4:**
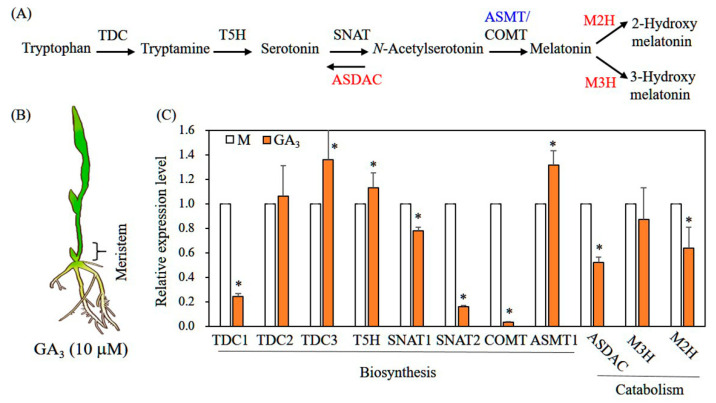
Expression levels of transcripts encoding melatonin biosynthetic and catabolic genes. (**A**) Genes involved in melatonin biosynthesis and catabolism in plants. (**B**) Schematic representation of rice seedlings treated with GA_3_ and the apical meristem region. (**C**) Quantitative reverse-transcription polymerase chain reaction (qPCR) analysis of various genes involved in melatonin synthesis and catabolism. Asterisks (*) denote significant differences from the mock control (M) (*p* < 0.05; ANOVA, followed by Tukey’s HSD post hoc tests). *TDC1*, tryptophan decarboxylase 1 (AK069031); *TDC2* (AK103253); *TDC3* (Os08g0140500); *T5H*, tryptamine 5-hydroxylase (AK071599); *SNAT1*, serotonin *N*-acetyltransferase 1 (AK059369); *SNAT2* (AK068156); *COMT*, caffeic acid *O*-methyltransferase (AK064768); *ASMT1*, *N*-acetylserotonin *O*-methyltransferase (AK072740); *ASDAC*, *N*-acetylserotonin deacetylase (AK072557); *M3H*, melatonin 3-hydroxylase (AK067086); *M2H*, melatonin 2-hydroxylase (AK119413); *ACT1*, actin 1 (Os03g50885).

Seven-day-old rice seedlings were challenged with 10 μM GA_3_ for 12 h; their meristematic tissues were separated and harvested for total RNA extraction (Figure 4B). To determine whether GA_3_ treatment altered the expression of genes responsible for melatonin synthesis and degradation, we performed qPCR gene expression analysis using *ACT1* as a reference gene (Figure 4A). Expression levels of melatonin biosynthetic genes tryptophan decarboxylase 3 (*TDC3*), tryptamine 5-hydroxylase (*T5H*), and *N*-acetylserotonin *O*-methyltransferase 1 (*ASMT1*) were elevated by GA_3_ treatment; expression levels of the biosynthetic genes *TDC1*, *SNAT1*, *SNAT2*, and caffeic acid *O*-methyltransferase (*COMT*) were downregulated (Figure 4C). *SNAT1* and *SNAT2* were downregulated in melatonin-treated rice seedlings, suggesting feedback regulation upon melatonin response [24]. Among catabolic genes, GA_3_ treatment inhibited the expression of *N*-acetylserotonin deacetylase (*ASDAC*) and melatonin 2-hydroxylase (*M2H*), whereas the expression of melatonin 3-hydroxylase (*M3H*) was not altered; the suppression of these two catabolic genes presumably facilitated melatonin production under GA_3_ treatment compared with the mock control. Because GA action occurs in meristem tissues, we applied qPCR analysis to meristematic tissues [25]. The expression profiles of genes related to melatonin biosynthesis and catabolism in rice upper leaves were not significantly altered in response to GA_3_ treatment (data not shown).

### 3.5. Decreased Melatonin Production in Transgenic Rice Plants Downregulating the GA Biosynthetic Gene GA3ox2

**Figure 5:**
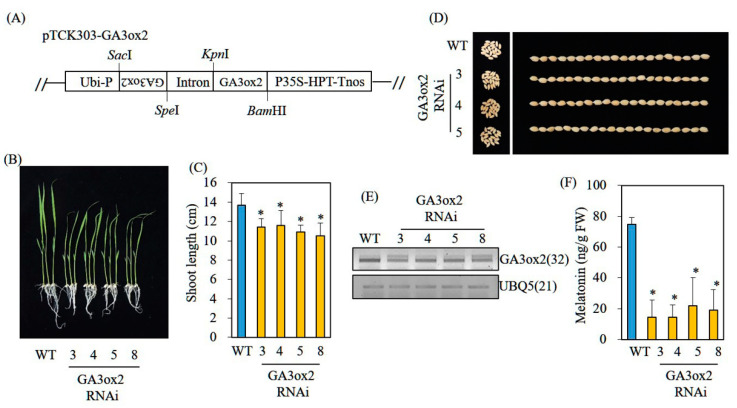
Structure of binary vector pTCK303-GA3ox2 and the generation of *GA3ox2*-suppressed transgenic rice plants. (**A**) Schematic diagram of the pTCK303:GA3ox2 binary vector. (**B**) Seven-day-old rice seedling phenotypes of wild-type (WT) and *GA3ox2* RNAi transgenic rice (T_2_). (**C**) Shoot length measurement. (**D**) Seed phenotype. (**E**) RT-PCR analysis results for WT and T_2_ lines. (**F**) Melatonin levels of WT and transgenic lines. Seven-day-old rice seedlings challenged with 0.5 mM cadmium for 3 days, then subjected to melatonin quantification. Asterisks (*) indicate significant differences from the WT (*p* < 0.05; ANOVA, followed by Tukey’s HSD post hoc tests). Numbers in parentheses indicate numbers of PCR cycles. GenBank accession numbers: Os01g0177400 (*GA3ox2*) and Os03g13170 (*UBQ5*). Ubi-P, maize ubiquitin promoter; P35S, 35 S cauliflower mosaic virus 35S promoter; HPT, hygromycin phosphotransferase; Tnos, nopaline synthase terminator.

To verify the involvement of GA in melatonin production in vivo, we generated transgenic rice plants downregulating the key GA biosynthetic gene GA3-oxidase 2 (*GA3ox2*), which catalyzes inactive GA_9_/GA_20_ into active GA_4_/GA_1_. Its knockout mutant, *D18*, is deficient in GA_1_ and has a dwarf phenotype [26]. Three independent homozygous transgenic RNAi lines downregulating rice *GA3ox2* were generated; the resulting *GA3ox2* RNAi lines showed semidwarf phenotypes (Figure 5A,C), whereas seed phenotypes of the *GA3ox2* RNAi lines were similar to the seed phenotypes of wild-type (WT) plants (Figure 5D). These phenotypic features were similar to the features of *d18* mutant rice [26]. *GA3ox2* mRNA levels were suppressed in three independent *GA3ox2* RNAi lines compared with the levels in the nontransgenic WT (Figure 5E). When these *GA3ox2* RNAi seedlings had been challenged with cadmium to induce melatonin production, WT produced 75 ng/g FW melatonin, whereas these RNAi lines produced threefold lower melatonin than that of the WT (Figure 5F). In the absence of cadmium treatment, WT rice produced about 0.3 ng/g FW melatonin, whereas *GA3ox2* RNAi lines produced half of the melatonin quantity in WT seedlings (data not shown). These data indicate that endogenous GA levels are functionally coupled to melatonin production as a positive melatonin synthesis-inducing factor. 

### 3.6. Decreased Melatonin Production in Transgenic Rice Plants Downregulating G-Protein Alpha (Gα)

**Figure 6:**
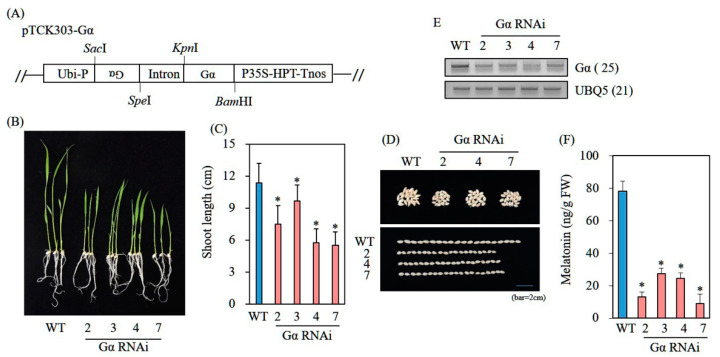
Structure of binary vector pTCK303-Gα and generation of *Gα*-suppressed transgenic rice plants. (**A**) Schematic diagram of pTCK303:Gα binary vector. (**B**) Seven-day-old rice seedling phenotypes of WT and *Gα* RNAi transgenic rice (T_2_). (**C**) Shoot length measurement. (**D**) Seed phenotype. (**E**) RT-PCR analysis results for WT and T_2_ lines. (**F**) Melatonin levels of WT and T_2_ lines. Seven-day-old rice seedlings challenged with 0.5 mM cadmium for 3 days and then subjected to melatonin quantification. Asterisks (*) indicate significant differences from WT (*p* < 0.05; ANOVA, followed by Tukey’s HSD post hoc tests). Numbers in parentheses indicate the numbers of PCR cycles. GenBank accession numbers: D38232 (*Gα*) and Os03g13170 (*UBQ5*). *Ubi-P*, maize ubiquitin promoter; *P35S*, 35 S cauliflower mosaic virus 35S promoter; *HPT*, hygromycin phosphotransferase; *Tnos*, nopaline synthase terminator.

**Figure 7:**
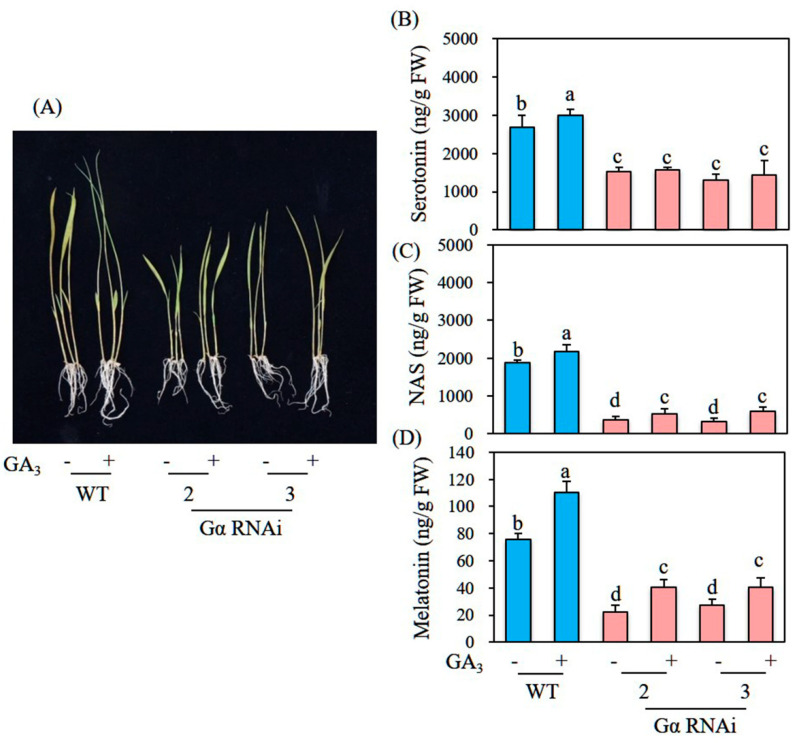
Effect of GA3 in the *Gα* RNAi lines. (**A**) Seedling phenotypes in response to GA_3_ (10 μM) treatment for 24 h, followed by treatment with 0.5 mM cadmium for 3 days. (**B**) Serotonin levels after GA_3_ and cadmium treatment. (**C**) *N*-Acetylserotonin levels after GA_3_ and cadmium treatment. (**D**) Melatonin levels after GA_3_ and cadmium treatment. Seven-day-old rice seedlings of WT and T_2_ homozygous *Gα* RNAi lines were used. Data are means of three replicates. Different letters denote significant differences (*p* < 0.05; ANOVA, followed by Tukey’s HSD post hoc tests).

*Gα* participates in a key GA signaling component; its mutant rice *d1* produces round dwarf grains [27,28]. To determine whether transgenic rice suppressing *Gα* exhibits less melatonin synthesis than that of its WT counterpart, we generated rice *Gα* RNAi transgenic lines. As observed in *Gα* mutant rice (*d1*) plants [28,29], *Gα* RNAi plants exhibited typical phenotypes characterized by round dwarf seeds (Figure 6A–E). When these *Gα* RNAi rice seedlings had been challenged with cadmium to induce melatonin production, four independent homozygous *Gα* RNAi rice seedlings produced melatonin at a mean of 20 ng/g FW, which was threefold less than the quantity produced by WT seedlings, indicating a positive effect of GA on melatonin production (Figure 6F). However, the GA_3_-induced melatonin increase was not abolished in these *Gα* RNAi rice seedlings compared with WT seedlings (Figure 7A,D). This result for *Gα* RNAi rice seedlings was similar to the phenotype of *d1* mutant rice, which does not completely lose GA sensitivity, suggesting the presence of a *Gα*-independent GA pathway [29]. In contrast, the induction of melatonin biosynthetic precursors serotonin but not *N*-acetylserotonin was abolished in these *Gα* RNAi rice seedlings by GA treatment; these precursors were induced in a similar pattern to melatonin induction upon GA treatment in WT seedlings (Figure 7B,C). The increase in serotonin is presumably attributable to the enhanced expression of *TDC3* upon GA treatment in WT seedlings; the increase in melatonin may have resulted from the combined effects of increased *TDC3* expression and decreased expression of catabolic genes such as *ASDAC* and *M2H*. 

## 4. Discussion

Melatonin biosynthesis is initiated from aromatic amino acid tryptophan in four steps catalyzed by tryptophan decarboxylase (TDC), tryptamine 5-hydroxylase (T5H), SNAT, and ASMT [13]. In plants, melatonin synthesis requires light, reactive oxygen species (ROS), and various photoreceptors (e.g., phytochromes and cryptochromes) [16,23,30]. Many biotic and abiotic stresses that cause ROS production can contribute to increased melatonin production in plants. Increased melatonin production in response to stresses counteracts the effects of ROS, either by directly scavenging ROS or by inducing an array of antioxidant enzymes (e.g., superoxide dismutase, ascorbate peroxidase, and glutathione S-transferase) [3,31,32]. For example, high light levels induced 38-fold higher melatonin production compared with low light in St. John’s wort [33]; a sixfold increase in melatonin synthesis was observed in barley roots in response to 10 mM hydrogen peroxide (H_2_O_2_) treatment [34]. Furthermore, bacterial and fungal pathogens induced melatonin synthesis in *Arabidopsis* [14] and cotton [35], respectively. 

Commensurate with melatonin induction in response to many stresses, the adverse effects of these stresses are mitigated by melatonin partly via crosstalk among various endogenous hormones and melatonin, which was presumably acquired during plant evolution after the advent of plant hormones [3]. The effects of melatonin on plant hormone regulation are broad and vary among plant species and stress factors. For example, exogenous melatonin treatment increased indole-3-acetic acid production in Chinese mustard [36] but decreased this production in *Arabidopsis* [37]. Contrasting effects of melatonin on other hormones (e.g., ABA and ethylene) were also observed in other plant species [38]. Two recent studies reported important discoveries regarding the inter-relationships of endogenous melatonin with endogenous hormone levels. In the *Arabidopsis SNAT2* knockout plant (*snat2*) showing a delayed flowering phenotype, it was found to decrease the expression of *ent*-kaurene synthase (*KS*), a key GA biosynthetic gene; this led to reduced GA levels [17]. In contrast, exogenous melatonin treatment suppressed *KS* expression, indicating contrasting effects of melatonin in vivo and in vitro. These data suggest that decreased GA levels in *Arabidopsis snat2* are indirectly caused by melatonin [17]. Similar to *Arabidopsis snat2*, transgenic rice downregulating rice *SNAT2* (*snat2*) exhibited decreased BR levels accompanied by BR-deficient erect leaf and dwarf phenotypes [24]. Because BR regulates GA levels [39], these data showing a GA decrease related to *Arabidopsis snat2* and a BR decrease related to rice *snat2* suggest that GA is closely associated with melatonin synthesis in plants.

**Figure 8:**
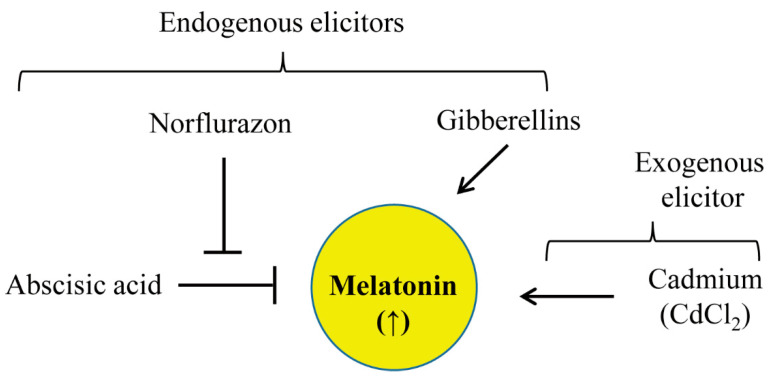
Proposed model of melatonin induction by endogenous hormones. GA induces melatonin production, whereas ABA inhibits melatonin production. Norflurazon, an ABA biosynthetic inhibitor, functions as a potent elicitor of melatonin production.

As predicted, GA had a positive effect on melatonin synthesis in rice plants, but the GA induction by melatonin was not observed (Figure 8). Generally, GA promotes growth via cell expansion and division in parallel with many other physiological functions including seed germination, flowering, and photomorphogenesis [40]. In particular, GA orchestrates these functions through the negative transcriptional repressor protein DELLA. GA triggers DELLA degradation, thereby releasing many active functions inhibited by DELLA; conversely, DELLA accumulation in the absence of GA inhibits growth, flowering, seed germination, and skotomorphogenesis, among other effects [40]. GA-mediated DELLA degradation leads to increased ROS, resulting in growth promotion and decreased stress tolerance; DELLA accumulation causes stress tolerance by decreasing ROS. A major question related to GA-induced melatonin synthesis is why GA induces melatonin, while melatonin does not induce GA. GA-induced growth promotion may be vulnerable to many adverse stresses that are counteracted by GA-induced melatonin synthesis because melatonin acts as a potent signaling molecule to protect plants from a diverse array of abiotic and biotic stresses [3]. Regarding the failure of melatonin-induced GA production, exogenous melatonin treatment does not induce *KS*, the key gene for GA synthesis [17]; this observation suggests that melatonin synthesis acts downstream of GA. The reduction of GA in melatonin-deficient *Arabidopsis snat2* was ascribed to decreased starch synthesis [4], which eventually led to decreased GA synthesis [41]. The potent inhibitory effects of ABA on melatonin biosynthesis are in sharp contrast to the effects of GA. Both ABA and GA exhibit robust effects on plant growth and development processes including seed germination, stem elongation, flowering, and seed development. However, GA and ABA have antagonistic effects: GA generally promotes these plant processes, while ABA inhibits them [42]. In parallel with the antagonistic effects of GA and ABA on many physiological functions, these two hormones antagonistically regulate melatonin biosynthesis: GA promotes melatonin biosynthesis, while ABA inhibits this process. 

To our knowledge, this study is the first to demonstrate that GA can induce melatonin production in rice seedlings and seeds. GA treatment onto mature rice plants grown in the field condition starting at 1 week after flowering affected neither flower development nor other growth parameters such as plant height. The direct involvement of GA in melatonin synthesis was verified by genetic evidence that GA biosynthetic (*GA3ox2* RNAi) and signaling mutants (*Gα* RNAi) result in severe decreases in melatonin production. Melatonin is a well-known health-promoting molecule with potent antioxidant activity that is positively involved in anti-inflammatory, antiaging, innate immunity enhancement, and anticancer activities [43]. GA is also widely used in the agricultural industry to promote crop productivity by inducing seedlessness in fruits (e.g., grapes) or increasing seed germination and fruit production in many plants [42,44]. Our results may contribute to the development of a practical approach for increasing melatonin in rice leaves and seeds through exogenous GA treatment; the resulting melatonin-rich seeds and plants may be used as functional foods for their health benefits.

## 5. Conclusions

In the present study, we reported the mechanistic basis for a practical approach to increase melatonin production in rice leaves and seeds through GA treatment; the resulting melatonin-rich seeds and plants may be used as functional foods for their health benefits. The role of GA as an endogenous elicitor of melatonin synthesis was verified in vivo using transgenic rice plants downregulating GA synthesis and signaling pathway. Transgenic rice plants downregulating GA3ox2 (GA biosynthesis) or Gα (signaling) synthesized less melatonin than that of wild-type plants. Our findings imply that plants or seeds with enriched melatonin levels can be produced through exogenous GA treatment under field conditions. These melatonin-rich agricultural products benefit human health through their aging prevention and antioxidant properties. 

## Data Availability

Data presented in this study are available within the article.
